# Occurrence of Polyamines in Foods and the Influence of Cooking Processes

**DOI:** 10.3390/foods10081752

**Published:** 2021-07-29

**Authors:** Nelly C. Muñoz-Esparza, Judit Costa-Catala, Oriol Comas-Basté, Natalia Toro-Funes, M. Luz Latorre-Moratalla, M. Teresa Veciana-Nogués, M. Carmen Vidal-Carou

**Affiliations:** 1 Departament de Nutrició, Ciències de l’Alimentació i Gastronomia, Facultat de Farmàcia i Ciències de l’Alimentació, Campus de l’Alimentació de Torribera, Universitat de Barcelona, Av. Prat de la Riba 171, 08921 Santa Coloma de Gramenet, Spain; nelly.munoz@ub.edu (N.C.M.-E.); jcostacatala@ub.edu (J.C.-C.); oriolcomas@ub.edu (O.C.-B.); mariluzlatorre@ub.edu (M.L.L.-M.); veciana@ub.edu (M.T.V.-N.); 2 Institut de Recerca en Nutrició i Seguretat Alimentària (INSA·UB), Universitat de Barcelona, Av. Prat de la Riba 171, 08921 Santa Coloma de Gramenet, Spain; 3 Xarxa d’Innovació Alimentària (XIA), C/Baldiri Reixac 4, 08028 Barcelona, Spain; 4 Universidad Internacional de Valencia, C/Pintor Sorolla 21, 46002 Valencia, Spain; natalia.toro@ub.edu

**Keywords:** polyamines, spermidine, spermine, putrescine, cooking processes, boiling, grilling, microwave, sous-vide

## Abstract

Dietary polyamines are involved in different aspects of human health and play an important role in the prevention of certain chronic conditions such as cardiovascular diseases and diabetes. Different polyamines can be found in all foods in variable amounts. Moreover, several culinary practices have been reported to modify the content and profile of these bioactive compounds in food although experimental data are still scarce and even contradictory. Therefore, the aim of this study was to evaluate the occurrence of polyamines in a large range of foods and to assess the effect of different cooking processes on the polyamine content of a few of them. The highest level of polyamines was found in wheat germ (440.6 mg/kg). Among foods of a plant origin, high levels of total polyamines over 90 mg/kg were determined in mushrooms, green peppers, peas, citrus fruit, broad beans and tempeh with spermidine being predominant (ranging from 54 to 109 mg/kg). In foods of an animal origin, the highest levels of polyamines, above all putrescine (42–130 mg/kg), were found in raw milk, hard and blue cheeses and in dry-fermented sausages. Regarding the influence of different domestic cooking processes, polyamine levels in food were reduced by up to 64% by boiling and grilling but remained practically unmodified by microwave and sous-vide cooking.

## 1. Introduction

Found in all living organisms, polyamines are nitrogenous low molecular weight substances characterized by the presence of two or more amino groups and are classed as spermidine (N-(3-aminopropyl)-1,4-butane diamine), spermine (N, N-bis(3-aminopropyl)-1,4-butane diamine) and putrescine (1,4-butanediamine). Their chemical structure confers notable stability to these compounds, which are capable of resisting acid and alkaline conditions and are highly soluble in hydroxyl solvents such as water and alcohol [1].

Polyamines participate in several biological processes, mainly cell proliferation and differentiation, protein synthesis, RNA transcription, the stabilization of negative charges of DNA and cell apoptosis and also possess antioxidant properties [2,3,4]. Multiple studies have been performed that support the involvement of polyamines in maintaining human health [5]. Due to their antioxidant and anti-inflammatory effects, polyamines play an important role in the prevention of chronic conditions such as cardiovascular diseases (i.e., hypertension, heart failure and myocardial fibrosis) and diabetes [6,7]. In addition, polyamines are associated with a protective effect against the aging process and have been described as potential lifespan-promoter compounds [8]. Spermidine can induce autophagy and reduce histone acetylation, which are critical processes for cellular homeostasis in aging [9]. Furthermore, a recent intervention study performed by Soda et al. [10] in a Japanese population showed that a long-term high intake of spermidine and spermine through the consumption of natto resulted in elevated blood spermine levels. These authors also report that an increased polyamine intake inhibits the pro-inflammatory state by decreasing the expression of LFA-1 (lymphocyte function-associated antigen) and suppressing aberrant gene methylation, which are related to the presence of age-associated chronic diseases [10]. Finally, it has been described that in certain situations that entail a rapid cell growth such as the neonatal stage and after surgery as well as in the elderly, polyamine requirements are higher [11,12,13].

**Figure 1:**
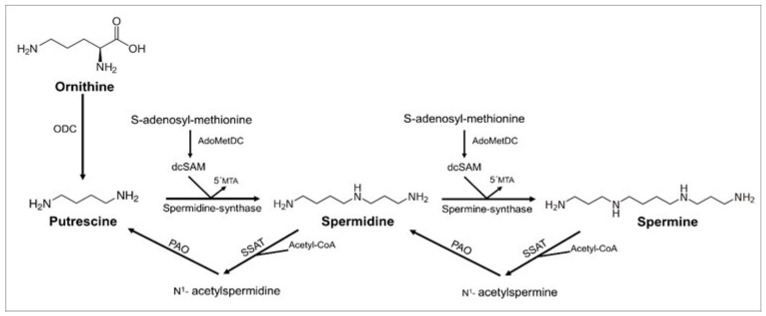
Synthesis and interconversion of polyamines. ODC: ornithine-decarboxylase; AdoMetDC: S-adenosyl-L-methionine-decarboxylase; dcSAM: decarboxylated S-adenosyl-methionine; 5’MTA: 5′-methylthioadenosine; SSAT: spermidine/spermine N1-acetyl-transferase; Acetyl-CoA: acetyl coenzyme-A; PAO: polyamine-oxidase. Adapted from Muñoz-Esparza et al. [15].

In the human organism, polyamine levels are mainly regulated by de novo synthesis (Figure 1). Briefly, putrescine is synthesized from the amino acid ornithine by the enzymatic action of ornithine decarboxylase. Putrescine is subsequently converted by spermidine synthase into spermidine, which is finally transformed to spermine in a reaction catalyzed by spermine synthase [14,15]. There is also a cyclical process of interconversion among polyamines that controls their turnover and regulates their intracellular homeostasis (Figure 1).

On the other hand, polyamines in the organism can also have an exogenous origin, mainly proceeding from food. Dietary polyamines are absorbed in the small intestine through transcellular or paracellular pathways and are distributed to the different tissues where they can be used directly by the cell or undergo interconversion [12,16]. Another potential source of polyamines is the intestinal microbiota. However, this contribution to the global pool of polyamines in the organism is still uncertain and more information is needed on the polyamine-forming capacity of the intestinal microbiota and the possible biosynthetic pathways [16,17].

In foods, spermidine, spermine and putrescine occur naturally, being present in animal and plant tissues [15,16,17,18]. High levels of putrescine may arise in several foods due to the amino acid decarboxylase activity of fermentative and/or spoilage microorganisms [19,20]. Similarly, although much less described, it has been postulated that spermidine and spermine could also have a bacterial origin, especially in fermented products [1,21]. Regarding individual polyamines, spermidine levels generally tend to be higher than those of spermine in foods of a plant origin and vice versa in those of an animal origin [1,15]. Most of the available data are from raw foods and only a few authors have considered the influence of cooking practices on the polyamine content. It has been reported that a few culinary practices could potentially have a modifying effect although experimental data are still scarce and even contradictory [18,22,23,24,25].

Bearing in mind the health benefits of polyamine intake and the potential interest of supplementing the diet with polyamines, it is of relevance to identify the main dietary sources of these compounds. Likewise, it is also necessary to know how technological or culinary treatments can affect the content of these bioactive compounds. Therefore, the aim of the present study was firstly to evaluate the occurrence of polyamines in different foods marketed in Spain and secondly to assess the effect of different cooking processes on the content of polyamines in a few of these foods.

## 2. Materials and Methods

### 2.1. Polyamine Content in Foods

Putrescine, spermidine and spermine contents were determined in 109 different food products retailed in Spain in the last 5 years. Overall, a total of 1406 samples were analyzed, belonging to eight different food categories: vegetables (27 food items, *n* = 292), fruit (22 food items, *n* = 130), cereals and derivatives (12 food items, *n* = 58), legumes (13 food items, *n* = 63), nuts and seeds (9 food items, *n* = 51), meat and derivatives (10 food items, *n* = 516), fish and seafood (9 food items, *n* = 188) and dairy products (7 food items, *n* = 108). The specific list of food items included in this study and the number of samples for each one are summarized in Supplementary Tables S1 and S2.

Polyamines were determined by ion-pair ultra-high performance liquid chromatography coupled to fluorometric detection (UHPLC-FL) as described by Latorre-Moratalla et al. [26]. Briefly, 5–10 g of a food sample, previously minced and homogenized, was mixed with 10 mL of 0.6 M perchloric acid (PanReac AppliChem GmbH, Darmstadt, Germany) on a magnetic stir plate for 20 min. Subsequently, the two phases were separated by centrifugation (20,000 rpm, 4 °C, 25 min) and the supernatant was filtered and collected in a 25 mL volumetric flask. This extraction process was repeated twice and the final volume of the extract was adjusted with 0.6 M perchloric acid. All samples were filtered through a GHP 0.22 μm filter (Waters Corp., Milford, MA, USA) and stored at 4 °C until the analysis. All determinations were done in triplicate. The chromatographic determination of the polyamines was accomplished using an Acquity UPLC BEH C18 1.7 µm reverse phase column (2.1 mm × 50 mm) (Waters Corp., Milford, MA, USA), followed by an online post-column derivatization with ortho-ophthaldehyde (Sigma-Aldrich, St. Louis, MO, USA) and spectrofluorometric detection (ex: 340 nm and em: 445 nm) (Waters Corp., Milford, MA, USA). The quantification of the polyamine content of the samples was carried out with the external standard method.

### 2.2. Effect of Different Cooking Processes on the Food Polyamine Content

In order to assess the effect of different cooking processes on the polyamine levels, thirteen food items with significant amounts of polyamines and which are usually consumed cooked were studied (i.e., mushrooms, asparagus, green beans, cauliflower, cabbage, broccoli, zucchini, spinach, pumpkin, chard, chicken meat, beef meat and pork meat). For this purpose, each food sample was divided homogeneously into five equal parts (100–120 g) and subjected to the different culinary processes: raw (control), boiling, grilling, microwave and sous-vide. Table 1 summarizes the specific conditions applied in each cooking process.

The polyamine content was determined following the analytical method described in the previous section. The water used for the boiling process was also analyzed for its polyamine content. Salt, oil or seasonings were not used in any cooking process. The results were expressed in dry matter in order to compare the effect of the different cooking methods. The moisture was determined according to the Official Method of the Association of Official Analytical Chemists, which consists of drying the samples (102 ± 2 °C) until a constant weight is reached [27].

### 2.3. Statistical Analysis

Polyamine contents are presented as mean values, standard deviation, minimum and maximum. A Student’s *t*-test was used to determine the significance of the influence of the cooking method on the polyamine content. The statistical analysis was performed with the IBM SPSS Statistics 25.0 statistical software package (IBM Corporation, Armonk, NY, USA). Values of *p* < 0.05 were accepted as significant.

## 3. Results and Discussion

### 3.1. Polyamine Content in Foods

**Figure 2:**
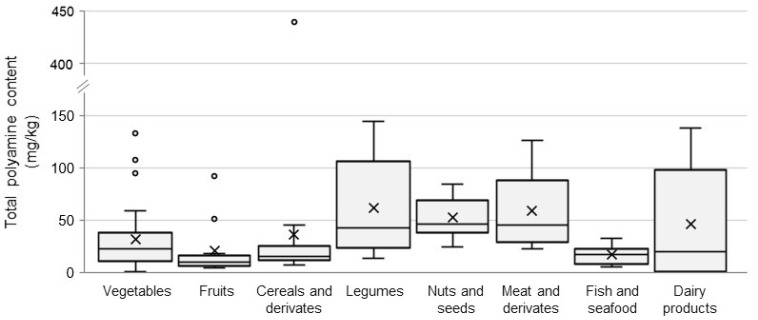
Distribution of the total polyamine content (mg/kg) by food category. The bottom and top of the box (interquartile range) are the percentile 25 and the percentile 75, respectively. The central line represents the median. Lines extending vertically from the boxes (whiskers) indicate variability outside the interquartile range. Outliers are plotted as circles.

Figure 2 shows the distribution of the total polyamine content in different food categories. Detailed data on the polyamine content for each foodstuff are provided in Supplementary Tables S1 and S2. Polyamines were found in all the foods although with a high variability. In general, the category of legumes showed the highest polyamine content, closely followed by dairy products and meat and derivatives (Figure 2). The variability in polyamine contents is reflected in the high interquartile ranges, calculated as the difference between percentile 75 and percentile 25, being particularly high in legumes (55.4 mg/kg) and dairy products (82.9 mg/kg). In contrast, fruit, cereals and fish and seafood showed less disparity in the polyamine content with interquartile ranges up to ten-fold smaller compared with the above-mentioned food categories.

**Figure 3:**
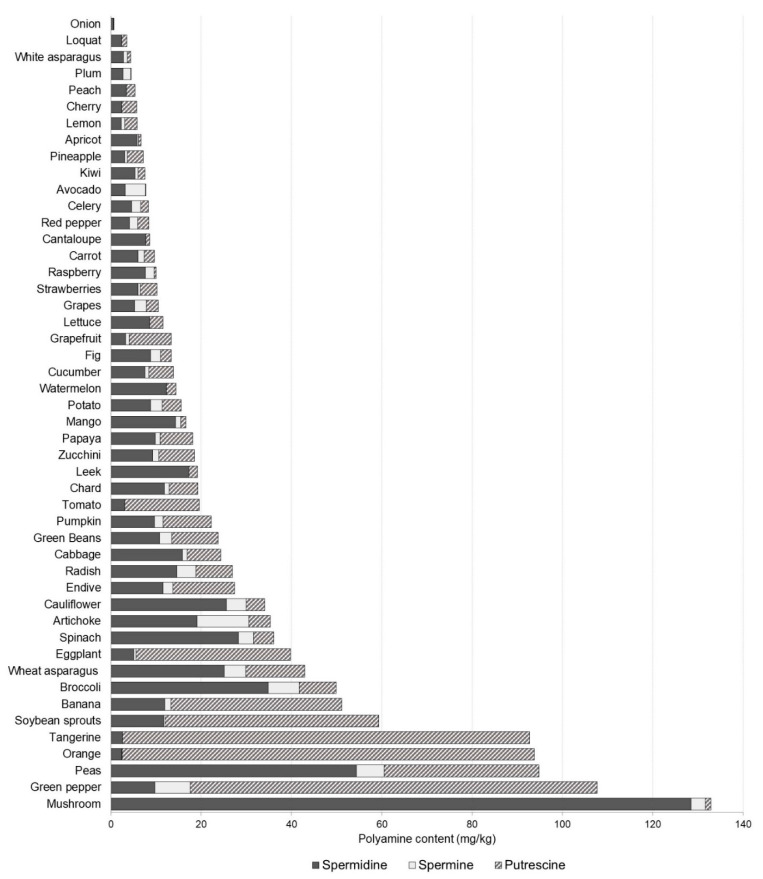
Polyamine profile and content (mg/kg) in vegetables and fruit.

Among vegetables and fruit, mushrooms, green peppers, peas and citrus fruit stand out for their high total polyamine content with the mean values being four-fold higher than in the other products from this category (Figure 3). Spermidine was the main polyamine in most vegetables and fruit, 90% of which contained between 2.4 mg/kg and 27.4 mg/kg. Mushrooms and peas stood out for particularly high contents of this polyamine with mean values of 128.50 mg/kg and 54.4 mg/kg, respectively. On the contrary, putrescine was the main polyamine in tomatoes, eggplants, bananas, soybean sprouts, green peppers and citrus fruit, representing 75–96% of their total polyamine content. The highest levels (a mean value of 90 mg/kg) were determined in green peppers and citrus fruit with very similar contents among samples. Similar results have been reported by other authors, suggesting that the accumulation of putrescine in these food types probably has a physiological origin [1,22,28]. In other vegetables such as spinach and peas, the high occurrence of putrescine has been linked with the presence of spoilage bacteria (i.e., *Enterobacteriaceae* and *Clostridium* spp.) [1,29].

**Figure 4:**
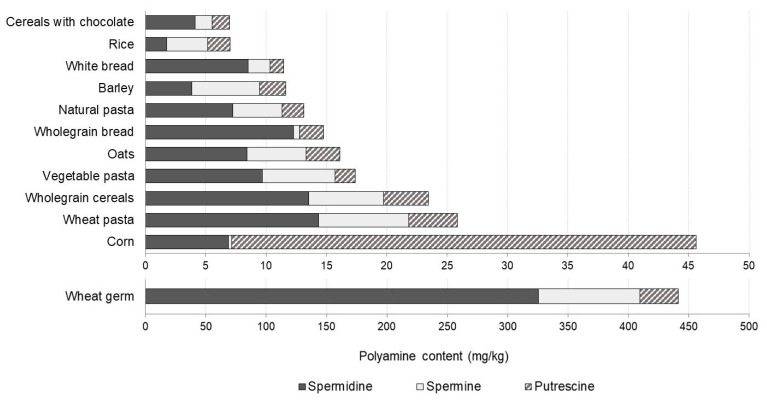
Polyamine profile and content (mg/kg) in cereals and derivatives.

In cereals and derivatives, the total polyamine levels were relatively low or moderate with mean values generally below 25 mg/kg (Figure 4). The exception was wheat germ, which had an outstandingly high polyamine content (440.6 mg/kg), far more than any other food item in the study. Among their many functions in plants, polyamines can act as growth factors, playing an important role during germination [30,31]. Thus, high levels of polyamines may be expected in all germinated foods such as those observed in wheat germ and, to a lesser extent, in soybean sprouts. Polyamines are reported to reach maximum levels after 48 h of germination and their accumulation may be influenced by conditions such as luminosity, temperature and humidity [32,33,34,35]. The predominant individual polyamine in practically all cereals and derivatives was spermidine. The exceptions were rice and barley, where the main polyamine was spermine, and corn, where putrescine prevailed, in agreement with reports by Zoumas-Morse et al. [36] and Bandeira et al. [37].

**Figure 5:**
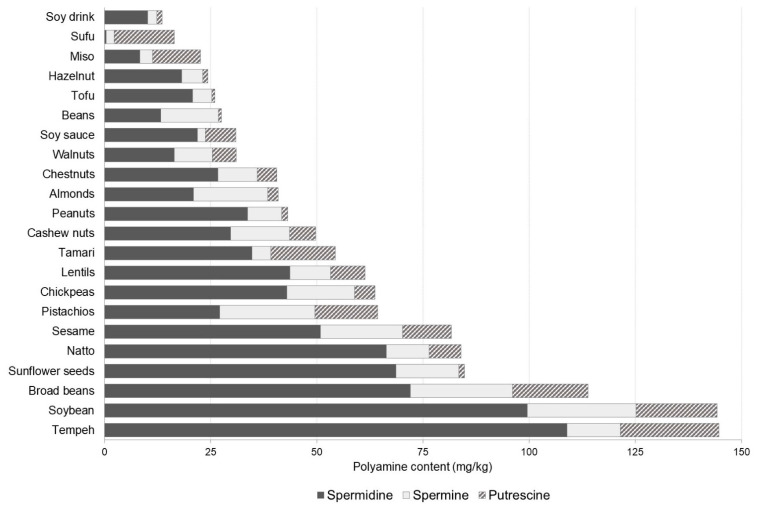
Polyamine profile and content (mg/kg) in legumes, nuts and seeds.

Polyamine contents in the different types of legumes, nuts or seeds were highly variable although the proportion of spermidine, spermine and putrescine was similar in practically all of them (Figure 5). Spermidine was the predominant polyamine in 86% of foods in this category with particularly high levels in certain legumes and derivatives (i.e., tempeh, soybeans, broad beans, natto, lentils and chickpeas) and seeds (sesame and sunflower seeds), reaching values of 43.0 mg/kg–108.9 mg/kg. The high amounts of putrescine in soy-fermented products (i.e., miso, sufu, soy sauce and tamari) could be related to the decarboxylase activity of both fermentative and/or spoilage bacterial strains [38]. Additionally, certain fermentation conditions such as temperature and duration may also have an impact on the accumulation of putrescine [38,39,40].

Finally, it is noteworthy that the occurrence of polyamines in all the above-mentioned plant-derived foods can be affected by the cultivar, geographical location, environmental factors and cultivation and/or harvesting conditions [25,32,41]. In this context, it has been extensively reported that polyamines play a key role in the plant response to various stressful environmental factors such as drought or the presence of extreme temperatures during the harvest period [31,42]. Specifically, it has been observed that putrescine levels in plants increase in response to stress during cultivation [35]. Likewise, several studies have shown that the exogenous application of polyamines, either pre-cultivation (i.e., soaking the seeds in water enriched with polyamines) or during cultivation through watering, can compensate for the negative effects of cold or drought, thus favoring the germination, growth or survival of plants [30,31,35,42]. These anti-stress properties of polyamines in plants could be attributed to their role in modulating morphological growth parameters, preserving the integrity of cell membranes and thus minimizing the growth inhibition caused by stress [43,44]. In addition, polyamines could increase the activity of several antioxidant enzymes present in plants, thus regulating oxidative stress caused by environmental factors [31].

**Figure 6:**
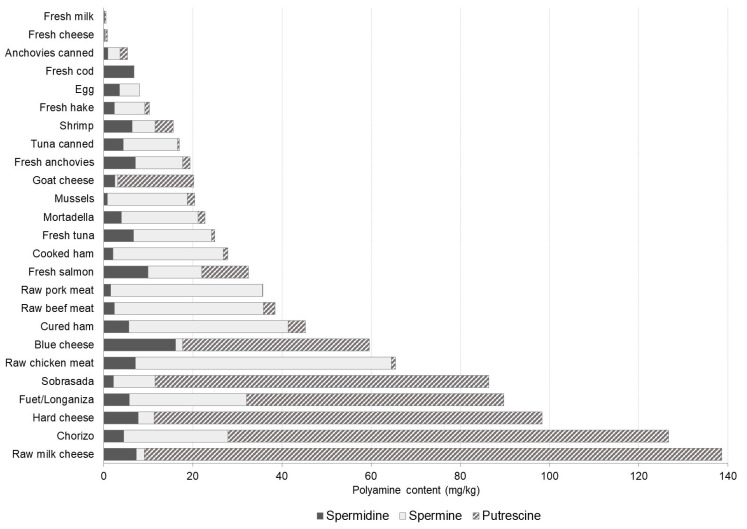
Polyamine profile and content (mg/kg) in meat, fish and dairy products.

In foods of an animal origin (meat, fish and dairy products), the total polyamine content was highly variable with values ranging from 0.15 mg/kg to 139 mg/kg (Figure 6). The highest levels were found in a few types of cheese (raw milk, hard and blue cheese) and dry-fermented sausages; products with an elevated content of putrescine arising from the aminogenic capacity of both fermentative microorganisms and Gram-negative spoilage bacterial strains [19,45]. Different authors have associated the use of raw meat of poor hygienic quality and/or improper production/storage conditions with a high accumulation of putrescine in fermented sausages [1,19]. Spermine was the predominant polyamine in the other animal-derived products, constituting 40% to 95% of the total polyamines in fresh meat, fresh fish, fish derivatives and cured and cooked meat products. Worth highlighting are the extremely low levels of any polyamine found in fresh milk and fresh cheese. They were also lower in yogurt compared with other fermented foods although slightly higher than those in milk. In eggs, only low levels of spermine and spermidine were determined, both in equal amounts. Despite available data on the polyamine content in milk, yogurt and eggs being very scarce, the levels reported in the literature are similar to those found in the present study [11,22,28].

### 3.2. Effect of Different Cooking Processes on Food Polyamine Content

Table 2 compares the polyamine content of thirteen food items with significant amounts of polyamines both raw and cooked by different culinary methods (i.e., boiling, grilling, microwave and sous-vide). This is the first time that the effect of these four cooking processes on the polyamine content in foods has been simultaneously assessed. In general, boiling and grilling reduced polyamine contents the most although this effect differed according to the type of food. Only cauliflower, broccoli and chicken meat did not show any polyamine loss after any of the culinary processes.

Boiling significantly decreased the polyamine content in most foods (9 out of 13) with losses ranging from 15 to 64% in comparison with raw foods depending on the polyamine. This decrease can be mainly attributed to their transfer to the boiling water, where polyamines were always recovered at a mean rate of 94%. This displacement of polyamines to water has also been reported by other authors [18,23,24,46,47,48]. Overall, chard, spinach, zucchini, pork and beef stood out for undergoing the highest reduction of polyamines especially putrescine (up to a 64% loss in chard). The high transfer of putrescine to boiling water can be explained by its greater solubility [15]. Moreover, polyamines are not homogenously distributed within a food product, which may account for the pronounced loss of putrescine in certain foods [46]. For example, it has been reported that asparagus accumulates higher concentrations of putrescine near the surface but not spermidine [49], which may explain the putrescine reduction in asparagus observed in the current study. Veciana-Nogués et al. [48] concluded that the putrescine content in spinach, cauliflower, chard and green beans was reduced by being transferred to the cooking water whereas boiling had no effect on other vegetables such as peppers and peas. Similarly, Eliassen et al. [22] and Bardocz et al. [50] reported no significant effect of boiling on the putrescine levels of vegetables such as carrot, broccoli, cauliflower and potato.

Grilling also resulted in notable losses of polyamines in practically the same foods as boiling. In general, more than a 20% reduction was observed in all polyamines with notably high spermine losses in cabbage (57%), spinach (39%) and zucchini (34%). The impact of grilling on the polyamine content has been scarcely studied to date and only for animal-derived foods. Available reports describe high losses of putrescine, spermidine and spermine in chicken and pork meat after grilling or roasting [18,23,46,47]. In the current study, the polyamine reduction in meat products was much lower and only observed in beef and pork. It has been hypothesized that the high temperatures reached during grilling (180 °C) may favor the so-called Maillard reaction by the interaction of the primary amino groups of polyamines with reducing sugars or, in the case of foods of an animal origin, with carbonyl compounds produced in the lipid oxidation pathway [18,51,52].

Microwave cooking caused a significant loss of polyamines only in zucchini, spinach, chard and beef but in considerably lower amounts in comparison with boiling and grilling. The affected polyamines were putrescine and/or spermidine. Microwave cooking can dehydrate food leading to the outflow of the most soluble polyamines and, accordingly, most of the lost polyamines were found in the water recovered from the cooking device. To compare the different foods, a standardized cooking time of five minutes was applied. The application of shorter times properly adapted to the nature of each food could minimize the leakage of water and hence the potential loss of polyamines.

Sous-vide is the French term used to designate the culinary method in which vacuumed foods are subjected to low temperatures for a long duration. One of the main features of sous-vide cooking is a high preservation of the organoleptic properties and compounds of raw food. Therefore, as expected, no significant loss of polyamines was observed for any food prepared with this technique and the minimal loss of water clearly prevented their leakage.

It should be noted that spinach was the only food in which amines other than polyamines were found. Specifically, a histamine content of 142 ± 6 mg/kg of dry matter was determined in raw spinach. In fact, spinach is one of the few plant foods to frequently contain histamine [20]. Comparable with their effect on putrescine, boiling and grilling of spinach caused significant histamine losses of nearly 60% and 25%, respectively. As both compounds are diamines, a similar behavior could be expected. These results are supported by a previous study, which also reported that adding salt when boiling had no effect on the high transfer of histamine from spinach to the cooking water [53].

## 4. Conclusions

In view of the reported benefits of polyamines for human health, including the prevention of certain chronic diseases, an increased intake of dietary polyamines is of potential importance. The great variability in the food polyamine content observed in this study indicates that the corresponding dietary recommendations should be issued considering each food product rather than general food categories. The best dietary sources of polyamines, especially spermidine, are wheat germ, soybeans and mushrooms. In future applications, these foods could be used to obtain plant extracts for food enrichment and/or polyamine supplements, which are especially recommended for certain stages of life with higher polyamine requirements.

Comparing domestic cooking processes revealed that boiling and grilling cause a significant reduction in the polyamine content in most of the tested foods, unlike microwave and sous-vide methods. Overall, the impact on polyamine levels depended not only on the type of food but also on the specific processing technique.

## Figures and Tables

**Table 1:** Specific conditions for each cooking process.

Cooking Method	Conditions	Cooking Time
Boiling	Sample was placed in 500 mL of boiling water.	15 min
Grilling	Sample was placed in the pan once it was hot (140 °C–160 °C).	7 min
Microwave	Sample was placed in a special microwave device made of silicone and cooked at 600 watts.	5 min
Sous-vide	Sample was vacuumed and sealed in a special plastic bag of polyamide and polyethylene with a thickness of 90 microns. It was subsequently immersed in water at 70 °C.	25 min

**Table 2:** Polyamine content (mean ± SD) in mg/kg of dry matter of foods subjected to different culinary processes: raw (control), boiling, grilling, microwave and sous-vide.

Food		Raw	Boiling	Grilling	Microwave	Sous-Vide
Mushroom	SD	1658.9 ± 7.0	* 1274.3 ± 19.1	1495.5 ± 18.4	1646.2 ± 30.4	1658.0 ± 31.9
	SM	39.6 ± 0.2	39.6 ± 2.2	35.9 ± 1.7	38.9 ± 2.3	39.1 ± 0.6
	PU	7.0 ± 0.1	6.9 ± 0.1	6.5 ± 0.2	6.7 ± 0.1	6.3 ± 0.4
Asparagus	SD	645.8 ± 40.8	645.7 ± 13.0	* 484.9 ± 20.2	641.8 ± 44.1	638.2 ± 4.9
	SM	125.8 ± 0.3	125.4 ± 0.1	* 91.0 ± 2.5	117.2 ± 8.1	122.8 ± 7.6
	PU	160.1 ± 3.5	* 121.0 ± 10.1	145.0 ± 7.4	143.9 ± 2.5	159.3 ± 1.2
Green beans	SD	185.6 ± 0.3	* 157.6 ± 0.1	* 156.1 ± 2.7	179.0 ± 0.4	174.1 ± 5.6
	SM	88.9 ± 2.0	* 73.3 ± 1.4	* 67.7 ± 1.1	80.0 ± 1.9	89.5 ± 2.2
	PU	12.9 ± 0.4	12.6 ± 0.4	12.5 ± 0.3	12.5 ± 0.2	12.4 ± 0.1
Cauliflower	SD	327.1 ± 9.0	325.8 ± 5.3	324.4 ± 8.3	320.3 ± 23.8	324.0 ± 35.9
	SM	66.6 ± 1.7	62.7 ± 2.1	65.5 ± 3.1	63.4 ± 1.5	64.4 ± 3.0
	PU	49.7 ± 0.6	48.4 ± 0.3	48.2 ± 2.1	47.2 ± 4.8	45.7 ± 4.3
Cabbage	SD	105.1 ± 1.8	105.3 ± 0.2	* 69.6 ± 0.2	96.1 ± 3.4	105.3 ± 0.3
	SM	36.4 ± 0.3	33.7 ± 7.2	* 15.9 ± 0.2	33.0 ± 0.3	35.9 ± 0.2
	PU	2.9 ± 0.1	2.37 ± 0.2	* 1.7 ± 0.1	2.8 ± 0.1	2.7 ± 0.1
Broccoli	SD	389.1 ± 23.9	382.6 ± 29.5	396.0 ± 17.4	389.9 ± 21.6	394.9 ± 4.0
	SM	77.2 ± 6.6	69.8 ± 4.0	73.8 ± 0.1	71.2 ± 0.7	72.1 ± 1.7
	PU	91.7 ± 5.6	91.7 ± 6.5	89.5 ± 4.0	85.9 ± 5.4	88.5 ± 0.9
Zucchini	SD	344.2 ± 3.4	* 298.0 ± 2.0	* 282.0 ± 1.7	* 301.7 ± 1.8	341.7 ± 12.0
	SM	62.8 ± 3.9	* 27.4 ± 4.9	* 41.6 ± 2.7	59.3 ± 1.4	51.0 ± 1.9
	PU	160.7 ± 5.6	* 133.1 ± 6.61	* 116.7 ± 1.1	* 123.2 ± 2.6	163.5 ± 12.9
Spinach	SD	330.2 ± 0.3	* 238.5 ± 20.89	* 236.1 ± 17.0	* 278.6 ± 1.1	317 ± 4.9
	SM	42.2 ± 2.5	* 31.5 ± 1.9	* 25.9 ± 1.0	38.8 ± 1.6	39.0 ± 0.5
	PU	40.8 ± 0.6	* 22.3 ± 4.7	* 31.4 ± 2.3	40.2 ± 1.7	38.1 ± 0.3
Pumpkin	SD	80.3 ± 0.6	* 66.6 ± 4.2	* 60.4 ± 1.1	78.1 ± 0.5	76.7 ± 0.6
	SM	840.3 ± 8.4	834.0 ± 10.4	* 664.8 ± 5.4	839.5 ± 0.8	833.2 ± 2.8
	PU	19.3 ± 0.5	* 15.1 ± 1.0	17.4 ± 2.5	19.8 ± 0.6	19.5 ± 0.1
Chard	SD	239.7 ± 11.4	* 178.0 ± 0.3	234.7 ± 2.7	* 196.6 ± 1.0	215.3 ± 14.5
	SM	28.4 ± 1.8	27.1 ± 1.0	26.0 ± 0.2	25.9 ± 0.6	28.1 ± 0.5
	PU	38.6 ± 1.7	* 13.7 ± 0.2	* 26.1 ± 0.6	35.1 ± 0.3	32.8 ± 0.7
Chicken meat	SD	1.5 ± 0.1	1.0 ± 0.1	1.4 ± 0.2	1.11 ± 0.0	1.2 ± 0.1
	SM	10.0 ± 0.9	8.2 ± 0.4	8.5 ± 0.0	8.4 ± 0.3	9.9 ± 0.4
	PU	0.4 ± 0.0	0.3 ± 0.0	0.3 ± 0.0	0.3 ± 0.1	0.4 ± 0.1
Beef meat	SD	9.3 ± 0.4	8.1 ± 0.1	* 7.5 ± 0.1	* 7.6 ± 0.5	8.6 ± 0.1
	SM	130.0 ± 9.5	* 94.4 ± 3.4	115.7 ± 0.4	120.8 ± 0.9	127.2 ± 9.5
	PU	10.4 ± 0.6	* 5.9 ± 0.1	* 7.1 ± 0.1	* 7.7 ± 0.3	9.4 ± 0.6
Pork meat	SD	9.4 ± 0.5	* 5.9 ± 0.1	* 7.7 ± 0.2	8.8 ± 0.3	9.0 ± 0.2
	SM	138.1 ± 1.0	* 89.0 ± 0.3	* 118.6 ± 3.4	137.6 ± 0.1	136.9 ± 0.8
	PU	0.4 ± 0.0	0.3 ± 0.1	0.4 ± 0.0	± 0.0	0.4 ± 0.0

* Significant differences in the polyamine content with respect to their raw food (*p* < 0.05).

## Supplementary Materials

The following are available online at https://www.mdpi.com/article/10.3390/foods10081752/s1, Table S1: Polyamine content (mg/kg) in food of a plant origin and Table S2: Polyamine content (mg/kg) in food of an animal origin.
