# Supplementary material for: Occurrence of Polyamines in Foods and the Influence of Cooking Processes
Source: Foods. 2021 Jul 29;10(8):1752. doi: 10.3390/foods10081752 (PMC8392025; doi:10.3390/foods10081752)
Supplement: Supplementary file 1 [file foods-10-01752-s001.zip › foods-1319688-supplementary.pdf]

Table S1. Polyamine content (mg/kg) in food of plant origin.

Food categories		Occurrence of polyamines (mg/kg)					
		Spermidine		Spermine		Putrescine	
	n	Mean ± SD	min – max	Mean ± SD	min – max	Mean ± SD	min – max
**Vegetables**							
Artichoke	5	19.1 ± 13.9	8.1 - 42.1	11.5 ± 9.8	4.9 - 28.6	4.8 ± 2.3	2.1 - 6.7
Broccoli	5	34.8 ± 2.1	33.3 - 36.3	6.9 ± 0.6	6.5 - 7.3	8.2 ± 0.5	7.9 - 8.6
Cabbage	3	15.9 ± 1.2	14.7 - 17.2	1.1 ± 1.8	Nd - 3.2	7.4 ± 0.6	7.0 - 8.1
Carrot	14	6.1 ± 2.2	4.3 - 11.2	1.4 ± 0.6	0.4 - 2.3	2.2 ± 2.0	0.4 - 8.9
Cauliflower	5	25.6 ± 5.7	19.1 - 32.9	4.4 ± 1.7	2.3 - 6.6	4.1 ± 2.1	1.6 - 7.1
Celery	3	4.6 ± 2.1	2.7 - 6.4	2.0 ± 0.9	1.3 - 2.6	1.8 ± 0.2	1.5 - 2.0
Chard	8	11.9 ± 2.7	8.3 - 15.4	1.0 ± 0.8	0.2 - 2.7	6.4 ± 3.2	2.4 - 11.9
Cucumber	10	7.6 ± 2.1	5.1 - 10.6	0.9 ± 1.0	0.2 - 2.9	5.4 ± 3.1	1.3 - 10.6
Eggplant	23	5.1 ± 1.9	2.5 - 8.0	0.5 ± 0.5	Nd - 1.4	34.3 ± 7.0	24.1 - 48.6
Endive	6	11.5 ± 2.4	10.9 - 14.4	2.2 ± 0.5	1.5 - 2.6	13.6 ± 2.0	11.3 - 15.9
Green Beans	12	10.8 ± 4.7	2.9 - 16.9	2.7 ± 1.9	0.7 - 7.2	10.3 ± 8.6	3.0 - 28.8
Green pepper	9	9.8 ± 8.3	Nd - 24.3	7.8 ± 5.7	Nd - 17.6	90.0 ± 41.7	11.7 - 149.0
Leek	3	17.3 ± 5.0	11.6 - 22.5	Nd	Nd	1.9 ± 0.6	1.2 - 2.4
Lettuce	2	8.6 ± 0.4	8.3 - 8.8	Nd	Nd	3.1 ± 1.2	2.2 - 3.9
Mushroom	11	128.5 ± 14.6	106.3 - 155.0	3.1 ± 0.4	2.4 - 3.8	1.3 ± 1.2	0.0 - 3.7
Onion	4	0.6 ± 0.3	0.2 - 0.7	Nd	Nd	0.1 ± 0.1	Nd - 0.1
Peas	9	54.4 ± 19.6	27.5 - 97.1	6.1 ± 2.4	3.1 - 9.9	34.3 ± 13.5	8.7 - 54.4
Potato	10	8.8 ± 1.9	5.6 - 12.3	2.6 ± 0.8	1.5 - 4.3	4.1 ± 3.1	1.1 - 11.7
Pumpkin	13	9.7 ± 3.6	1.8 - 15.0	1.9 ± 2.0	0.5 - 6.9	10.7 ± 6.8	3.0 - 24.2
Radish	5	14.7 ± 12.4	0.9 - 28.2	4.2 ± 4.8	1.5 - 12.3	8.1 ± 4.2	3.8 - 13.1
Red pepper	8	4.1 ± 0.7	3.1 - 5.0	1.8 ± 0.8	0.6 - 2.7	2.4 ± 2.2	0.6 - 5.4
Spinach	18	28.2 ± 9.7	15.6 - 53.0	3.3 ± 1.9	Nd - 8.9	4.5 ± 2.5	0.1 - 9.2
Soy sprouts	3	11.2 ± 2.1	10.5 - 11.7	0.5 ± 0.4	0.3 - 0.9	44.7 ± 3.2	41.1 - 47.4
Tomato	53	3.0 ± 1.4	2.9 - 7.9	0.1 ± 0.1	0.7 - 0.7	16.5 ± 6.9	6.3 - 35.6
Wheat asparagus	5	25.1 ± 7.4	14.1 - 32.5	4.8 ± 1.3	2.6 - 5.7	13.1 ± 2.8	8.6 - 16.3
White asparagus	4	2.8 ± 0.5	2.4 - 3.3	0.8 ± 0.2	0.5 - 1.0	0.8 ± 0.2	0.5 - 1.0
Zucchini	41	9.2 ± 2.9	5.4 - 14.9	1.4 ± 1.5	0.2 - 6.1	7.9 ± 4.1	2.7 - 24.8
**Fruits**							
Apricot	4	5.9 ± 1.6	4.2 - 7.7	0.2 ± 0.4	Nd - 0.9	0.6 ± 0.3	0.3 - 1.0
Avocado	5	3.2 ± 3.3	0.2 - 6.7	4.5 ± 2.5	2.0 - 7.9	0.1 ± 0.1	Nd - 0.3
Banana	8	11.9 ± 2.9	7.6 - 15.8	1.3 ± 1.0	Nd - 2.8	38.0 ± 8.3	25.5 - 49.5
Cantaloupe	3	7.7 ± 0.9	6.8 - 8.7	Nd	Nd	0.8 ± 0.3	0.6 - 1.1
Cherry	2	2.4 ± 0.2	2.3 - 2.5	Nd	Nd	3.4 ± 0.1	3.4 - 3.5
Fig	2	8.8 ± 0.4	8.6 - 9.1	2.2 ± 0.3	2.0 - 2.4	2.4 ± 0.3	2.2 - 2.6
Grapefruit	9	3.3 ± 1.8	1.1 - 5.4	0.7 ± 1.4	Nd - 3.5	9.4 ± 11.9	Nd - 28.0
Grapes	10	5.3 ± 2.6	Nd - 9.7	2.6 ± 0.1	2.4 - 2.7	2.7 ± 0.3	1.0 - 4.3
Kiwi	10	5.4 ± 1.1	2.7 - 6.4	0.7 ± 0.6	Nd - 1.5	1.5 ± 0.5	0.5 - 2.2
Lemon	5	2.3 ± 0.6	1.9 - 3.2	0.8 ± 0.7	Nd - 1.6	2.8 ± 1.1	1.2 - 3.7
Loquat	2	2.4 ± 0.2	2.3 - 2.5	Nd	Nd	1.2 ± 0.3	1.0 - 1.3
Mango	5	14.4 ± 4.3	10.3 - 19.1	1.2 ± 1.6	Nd - 3.0	1.1 ± 0.7	0.6 - 2.3
Orange	12	2.4 ± 0.9	1.5 - 4.2	0.1 ± 0.3	Nd - 1.1	91.2 ± 41.7	11.3 - 151.1
Orange juice	5	5.2 ± 0.6	1.6 - 5.8	3.6 ± 3.1	Nd - 5.4	41.9 ± 9.2	35.1 - 57.3

Papaya	6	9.9 ± 4.5	6.1 - 15.9	1.0 ± 1.2	Nd - 2.4	7.3 ± 5.8	Nd - 12.5
Peach	2	3.5 ± 0.9	2.8 - 4.1	Nd	Nd	1.9 ± 0.1	1.8 - 2.0
Pineapple	2	3.1 ± 0.1	3.0 - 3.2	0.6 ± 0.3	0.3 - 0.8	3.5 ± 0.1	3.4 - 3.6
Plum	2	2.7 ± 0.3	2.5 - 2.9	1.7 ± 2.5	Nd - 3.5	0.1 ± 0.1	Nd - 0.1
Raspberry	4	7.6 ± 1.3	6.3 - 9.4	2.0 ± 1.6	Nd - 3.7	0.5 ± 0.5	Nd - 1.0
Strawberries	9	6.0 ± 1.6	4.6 - 9.9	0.5 ± 0.7	Nd - 1.6	3.8 ± 1.5	2.0 - 6.4
Tangerine	21	2.6 ± 1.6	0.2 - 6.2	Nd	Nd	90.2 ± 36.6	12.3 - 173.8
Watermelon	2	12.3 ± 0.1	12.2 - 12.4	Nd	Nd	2.1 ± 0.0	2.1 - 2.2
Cereals and derivates							
Wheat germ	2	325.5 ± 3.0	323.3 - 327.6	84.2 ± 0.3	84.0 - 84.4	31.6 ± 0.4	31.4 - 31.9
Barley	2	3.9 ± 2.0	2.5 - 5.3	5.6 ± 2.0	4.2 - 7.0	2.2 ± 1.6	1.1 - 3.3
Oats	5	8.4 ± 4.7	3.3 - 12.2	4.9 ± 1.0	3.5 - 5.8	2.8 ± 2.0	0.4 - 4.3
Rice	5	1.8 ± 1.0	0.7 - 3.0	3.4 ± 1.1	2.5 - 4.8	1.9 ± 0.5	1.4 - 2.4
Corn	5	6.8 ± 6.5	1.1 - 14.1	0.3 ± 0.4	Nd - 0.9	38.4 ± 9.5	30.5 - 54.8
White bread	6	8.5 ± 6.7	3.0 - 17.8	1.8 ± 1.6	Nd - 3.6	1.1 ± 0.5	0.3 - 1.7
Wholegrain bread	6	12.3 ± 10.3	3.6 - 35.8	0.5 ± 0.9	Nd - 2.2	2.0 ± 1.5	Nd - 4.3
Natural pasta	5	7.3 ± 1.5	2.0 - 5.1	4.1 ± 1.2	2.0 - 5.1	1.8 ± 1.7	Nd - 3.1
Wheat pasta	5	14.4 ± 4.0	9.9 - 20.82	7.5 ± 1.9	5.9 - 10.3	4.0 ± 0.8	3.0 - 2.6
Vegetable pasta	5	9.7 ± 3.5	6.3 - 14.5	6.0 ± 1.6	4.3 - 7.9	1.9 ± 0.8	0.8 - 2.5
Whole grain cereals	6	13.5 ± 7.8	0.9 - 25.0	6.2 ± 2.7	1.6 - 2.7	3.7 ± 1.6	1.5 - 6.0
Cereals with chocolate	6	4.1 ± 2.2	0.5 - 6.3	1.4 ± 1.8	Nd - 4.7	1.4 ± 0.9	0.8 - 3.1
Legumes							
Beans	5	13.3 ± 1.5	11.0 - 14.5	13.6 ± 4.5	5.1 - 17.2	0.7 ± 0.7	0.4 - 2.0
Broad beans	10	72.0 ± 3.9	54.6 - 94.2	24.0 ± 3.8	18.9 - 30.8	17.9 ± 0.4	17.3 - 18.0
Chickpeas	5	43.0 ± 37.3	15.3 - 85.4	15.9 ± 14.8	4.3 - 32.4	4.8 ± 3.1	0.9 - 8.9
Lentils	7	43.7 ± 41.7	14.5 - 107.4	9.5 ± 5.1	5.1 - 18.4	8.2 ± 8.4	2.0 - 21.8
Soybean	2	99.6 ± 3.5	97.1 - 102.0	26.6 ± 7.8	20.1 - 31.1	19.1 ± 4.4	15.9 - 22.2
Soy drink	8	10.1 ± 0.5	9.1 - 11.0	2.2 ± 0.6	1.6 - 3.2	1.3 ± 0.4	0.7 - 2.1
Tofu	8	20.8 ± 6.3	3.4 - 30.2	4.5 ± 3.0	Nd - 8.1	0.8 ± 0.6	Nd - 1.5
Natto	3	66.4 ± 9.2	56.9 - 75.2	10.1 ± 1.0	9.2 - 11.2	7.4 ± 1.9	5.8 - 9.5
Tempeh	3	108.9 ± 13.7	97.3 - 124.0	12.6 ± 8.3	6.1 - 21.9	23.2 ± 7.0	17.5 - 31.1
Tamari	3	34.8 ± 4.6	29.5 - 38.0	4.4 ± 2.1	2.8 - 6.8	15.3 ± 2.0	13.1 - 17.1
Miso	3	8.4 ± 1.3	7.5 - 9.9	2.9 ± 0.6	2.5 - 3.5	11.4 ± 7.8	2.7 - 17.8
Sufu	3	0.5 ± 0.8	Nd - 1.4	1.8 ± 0.8	0.9 - 2.2	14.2 ± 4.7	9.3 - 18.8
Soy sauce	3	21.9 ± 1.7	19.9 - 22.9	1.9 ± 0.3	1.5 - 2.1	7.2 ± 0.4	7.0 - 7.6
Nuts and seeds							
Almonds	7	21.0 ± 4.9	14.4 - 25.6	17.5 ± 5.0	11.4 - 23.9	2.5 ± 1.2	Nd - 4.4
Cashew nuts	2	29.7 ± 4.7	26.3 - 33.0	14.0 ± 0.3	13.7 - 14.2	6.1 ± 0.7	5.7 - 6.6
Chestnuts	5	26.7 ± 7.1	14.2 - 31.5	9.2 ± 4.2	2.9 - 13.7	4.6 ± 1.7	2.1 - 6.9
Hazelnut	9	18.2 ± 6.9	9.0 - 29.5	4.9 ± 2.3	2.0 - 9.3	1.2 ± 1.1	Nd - 3.2
Peanuts	8	33.7 ± 13.4	22.6 - 58.3	8.0 ± 2.8	5.2 - 12.5	1.4 ± 1.8	0.2 - 5.0
Sunflower seeds	5	68.7 ± 58.0	14.3 - 140.6	14.7 ± 8.5	6.5 - 23.4	1.4 ± 0.8	0.4 - 2.0
Pistachios	7	27.1 ± 7.4	14.9 - 35.5	22.3 ± 13.7	10.8 - 50.8	14.8 ± 14.0	4.3 - 39.5
Sesame	2	50.9 ± 2.6	49.1 - 52.7	19.3 ± 0.9	18.7 - 19.9	11.5 ± 0.5	11.2 - 11.5
Walnuts	6	16.4 ± 6.6	6.2 - 23.8	9.0 ± 3.3	6.3 - 13.6	5.6 ± 4.2	2.8 - 13.8

Nd- not detected; All samples were analyzed in duplicate.

Table S2. Polyamine content (mg/kg) in food of animal origin.

Food categories		Occurrence of polyamines (mg/kg)					
		Spermidine		Spermine		Putrescine	
	n	Mean ± SD	min – max	Mean ± SD	min – max	Mean ± SD	min – max
**Meat and derivates**							
Raw beef meat	5	2.5 ± 0.3	2.4 - 2.6	26.6 ± 8.0	22.1 - 33.4	2.0 ± 0.8	1.6 - 2.7
Raw pork meat	5	1.6 ± 0.6	0.9 - 2.6	34.1 ± 2.7	30.7 - 38.1	0.1 ± 0.2	0.0 - 0.5
Raw chicken meat	5	7.2 ± 2.5	4.5 - 11.3	57.3 ± 4.5	51.0 - 63.3	1.0 ± 0.6	1.0 - 29.8
Cured ham	23	5.7 ± 1.0	4.1 - 7.3	35.8 ± 8.2	25.0 - 62.1	3.9 ± 4.0	Nd - 17.4
Cooked ham	20	2.1 ± 0.6	1.4 - 3.5	24.8 ± 17.0	6.0 - 87.8	1.0 ± 2.8	Nd - 12.4
Mortadella	20	4.0 ± 2.3	1.0 - 8.9	17.2 ± 7.5	8.0 - 32.2	1.6 ± 1.7	Nd - 5.7
Chorizo	107	4.6 ± 5.5	0.0 - 32.1	23.3 ± 17.0	Nd - 87.2	98.8 ± 115.2	Nd - 471.5
Fuet/Longaniza	305	5.8 ± 6.1	Nd - 32.7	26.3 ± 25.9	Nd - 224.2	57.7 ± 92.6	Nd - 537.1
Sobrasada	12	2.2 ± 2.0	Nd - 7.0	9.3 ± 6.2	Nd - 17.8	74.8 ± 141.6	Nd - 500.6
Egg	14	3.6 ± 1.5	Nd - 4.5	4.5 ± 1.7	0.3 - 5.3	Nd	Nd
**Fish and seafood**							
Fresh tuna	20	6.7 ± 3.7	1.2 - 11.7	17.5 ±11.9	1.1 - 37.0	0.7 ± 1.2	Nd - 4.9
Tuna canned in oil	37	4.5 ± 3.0	1.5 - 19.5	12.1 ± 6.58	2.2 - 35.2	0.5 ± 0.7	Nd - 2.2
Fresh hake	88	2.5 ± 1.9	Nd - 8.2	6.8 ± 2.8	Nd - 13.5	1.0 ± 2.1	Nd - 11.0
Fresh salmon	5	10.0 ± 3.2	7.0 - 15.2	12.0 ± 24.0	Nd - 54.8	10.5 ± 178.0	2.6 - 400.0
Fresh cod	5	6.8 ± 1.6	5.0 - 8.9	Nd	Nd	Nd	Nd
Fresh anchovies	10	7.1 ± 2.5	4.0 - 11.9	10.6 ± 3.0	7.0 - 15.4	1.7 ± 1.7	Nd - 4.9
Anchovies canned	16	1.0 ± 0.4	0.4 - 1.7	2.7 ± 0.7	1.2 - 4.3	1.7 ± 1.8	Nd - 5.7
Mussels	3	0.9 ± 0.2	0.7 - 1.1	18.0 ± 1.8	16.1 - 19.7	1.6 ± 0.2	1.4 - 1.8
Shrimp	4	6.4 ± 1.8	4.0 - 8.4	5.2 ± 0.2	4.9 - 5.4	4.1 ± 3.8	1.8 - 9.8
**Dairy products**							
Fresh milk	3	0.1 ± 0.1	Nd - 0.2	Nd	Nd	0.1 ± 0.2	Nd - 0.3
Yogurt	5	1.1 ± 0.8	0.5 - 1.8	0.3 ± 0.4	Nd - 0.5	2.0 ± 2.0	Nd - 4.1
Fresh cheese	20	0.3 ± 0.3	Nd - 0.8	0.2 ± 0.3	Nd - 1.1	0.4 ± 0.8	Nd - 3.1
Raw milk cheese	20	7.4 ± 12.9	Nd - 39.4	1.7 ± 5.3	Nd - 21.1	129.5 ± 212.7	Nd - 666.9
Blue cheese	20	16.2 ± 15.9	Nd - 68.9	1.5 ± 4.2	Nd - 18.9	41.9 ± 72.7	Nd - 252.3
Hard cheese	20	7.8 ± 10.3	Nd - 41.9	3.5 ± 5.6	Nd - 18.1	87.0 ± 151.7	Nd - 610.5
Goat cheese	20	2.5 ± 3.7	Nd - 14.3	0.6 ± 1.1	Nd - 3.5	17.1 ± 40.9	Nd - 184.0

Nd- not detected; All samples were analyzed in duplicate.
